# Understanding mental health challenges and associated risk factors of post-natural disasters in Bangladesh: a systematic review

**DOI:** 10.3389/fpsyg.2024.1466722

**Published:** 2024-12-16

**Authors:** Md. Zawadul Karim, Md. Al-Mamun, Maliha Azad Eva, Md. Hazrat Ali, Abul Kalam, Nurul Islam Uzzal, Pranto Kumer Das

**Affiliations:** ^1^Department of Psychology, Bangabandhu Sheikh Mujibur Rahman Science and Technology University, Gopalganj, Bangladesh; ^2^BRAC Institute of Governance and Development (BIGD), BRAC University, Dhaka, Bangladesh; ^3^Mass Communication and Journalism Discipline, Khulna University, Khulna, Bangladesh; ^4^Department of Genetic Engineering and Biotechnology, Shahjalal University of Science and Technology, Sylhet, Bangladesh; ^5^Department of Sociology, Bangabandhu Sheikh Mujibur Rahman Science and Technology University, Gopalganj, Bangladesh

**Keywords:** mental health, natural disasters, risk factors, systematic review, Bangladesh

## Abstract

**Background and objectives:**

Natural disasters are harmful occurrences caused by the Earth's geological and meteorological processes. Bangladesh is recognized as one of the country's most vulnerable to natural disasters. Therefore, the people of Bangladesh remain at high risk of natural disasters. However, no study has been conducted in Bangladesh to provide an overall perspective on mental health issues that arise after natural disasters. Thus, this systematic review aims to identify all mental health issues and related risk factors brought on by natural disasters in Bangladesh.

**Study design:**

Systematic review.

**Methods:**

Between April to May 2024, a systematic search was conducted across many databases, including PubMed, following the PRISMA guideline. Ultimately, 17 publications were included in this study.

**Results:**

The findings reveal that depression, anxiety, stress, suicidal ideation, post-traumatic stress disorder, major depressive disorder, generalized anxiety disorder and sleep disturbances are found as major risk factors for natural disasters. Prevalence of depression, anxiety and, stress, suicidal ideation ranges from 16.3% to 92.71%, 6% to 92.19%, 32.41% to 58%, 10.9% to 57.5% among people as consequences of natural disasters like heatwaves, cyclones, flood, river bank erosion etc. There are some associated risk factors which provoke prominent mental health problems, including (i) Demographic factors (age, gender, marital status, number of children, lower education, living location, living infrastructure, family conflicts, family member death, land for cultivation, loss of domestic animals), (ii) socio-economic factors (environmental settings, social support, disaster warning system, displacement, food crisis, financial support, income loss, vulnerable livelihoods) (iii) behavior and health-related factors (behavior pattern, physical injury during natural disaster, physical disability), (iv) other factors (increased temperature, living together, lower social class). People affected by natural disasters are often viewed in terms of their physical damage, while their mental health is always ignored.

**Conclusion:**

Calculative measures are needed to create an overall picture of the effect of natural disasters on people's mental health in Bangladesh. Therefore, the government needs to consider establishing potential measures to lessen the impact of natural disasters on people's mental health.

## 1 Background

*United Nations International Strategy* for Disaster Reduction, disaster is defined as “a substantial disturbance of a community's or society's regular operations that results in extensive losses and effects to people, property, or the environment that are more than what the impacted community can manage with its own resources” [United Nations International Strategy for Disaster Reduction (UNISDR), 2009]. According to the World Health Organization (2002), disasters disrupt normal conditions of existence and typically cause a level of suffering that exceeds the capacity of affected communities to adjust. A natural disaster is a catastrophic occurrence that happens on a large scale as a result of Earth's own geological and meteorological processes. Human casualties, physical harm, and material destruction are common outcomes of natural disasters (Saeed and Gargano, 2022). An increase in both the frequency and severity of catastrophes, particularly for countries with fewer resources, may pose the biggest threat to global health in the 21st century (Smith et al., 2022). Among the most prevalent and incapacitating mental illnesses worldwide are anxiety, despair, schizophrenia, stress, and drug misuse. According to the World Health Organization (2001), an estimated 12.0% of the global disease burden and 30.8% of the disability years were attributed to mental illness. Whiteford et al. (2013) found that this load increased by 37.6% between 1990 and 2010. Eliminating risk factors, such as the prevalence of gaps in mental health care following disasters, is, thus, a primary goal of public health policy.

Bangladesh is one of the world's most vulnerable nations due to its geographical and geophysical position, which puts it at increasing risk from the consequences of climate change. Both the IPCC 2011 and the Global Climate Risk Index 2020 place Bangladesh at number seven on the list of nations most impacted by climate change (Islam et al., 2021). As per CRI estimates, for the years 2000–2019, Bangladesh had the ninth-highest number of fatalities caused by disasters globally, and it placed seventh among countries hit by natural disasters (Eckstein et al., 2021). Because of its location, it is prone to catastrophic events that happen again and again (Karim and Mimura, 2008). Natural catastrophes in Bangladesh and other nations can be caused by cyclones, heavy rainfall, flooding, river bank erosion, and the possibility of sea level rise (Ali et al., 2019). The nation has 75 significant cyclones and floods reported in the last 100 years, which is a highly concerning fact. At least 1,200 kilometers of riverbank erosion occurs every year, which is also quite concerning (Ahmed et al., 2015). Consequently, about 30 million individuals in Bangladesh are at risk of flooding, river bank erosion, cyclones, and other similar disasters (Chowdhury et al., 2020). For instance, the devastating cyclone Amphan killed 25 people and affected over one million in nine coastal regions of Bangladesh (Kumar et al., 2021).

Numerous studies have shown a direct or indirect correlation between disasters and people's psychological wellbeing (Fritze et al., 2008). Most people have some kind of mental health concern, from mild to severe, after a disaster (World Health Organization, 2019). Both the economy and public health suffer greatly as a result of catastrophic natural disasters like floods. Though they may quickly get over physical injuries, survivors often struggle with mental health issues such as substance abuse, domestic violence, PTSD, depression, anxiety, insomnia, and adjustment disorders. Survivors may also overcome and recover completely from physical injuries (Beaglehole et al., 2018; Guo et al., 2017; Fernandez et al., 2015).

Nahar et al. (2014) found that among Bangladeshi cyclone Sidr survivors from 2007, 25% had PTSD, 16% had major depressive disorder, 15% had mixed anxiety, and 15% had somatoform disorder. Furthermore, 15% suffer from depression. When a natural disaster levels a person's home, it can be one of the most harrowing experiences that can cause lasting psychological damage. Depression and PTSD, two of the most common mental health issues, are associated with this traumatic event. Reviewing the mental health issues of Mexican flood victims in 1999, researchers found that many of them were still dealing with depression 6 months after the tragedy (Norris et al., 2004). As well as wreaking havoc on crops and buildings, natural catastrophes can devastate economies, endanger lives, and have little warning before they strike. Some survivors report experiencing mental instability as a result of these impacts (Dai et al., 2017). Many people's mental health can take a hit after experiencing stress, loss, or despair as a result of sudden weather disasters (Berry et al., 2018).

Bangladesh often experiences natural disasters. It is vulnerable to a wide range of natural disasters, including floods, cyclones, heat waves, river bank erosion, and more. People who have lived through natural disasters have shown an extraordinary degree of susceptibility to such events. People whom natural catastrophes have impacted often acquire mental health concerns. There has been a dearth of current research into the effects of natural catastrophes on mental health in various regions of Bangladesh, leading researchers to anticipate a plethora of mental health issues and their corresponding risk factors. The total prevalence of mental health issues in the aftermath of natural catastrophes in Bangladesh has not been studied. The only thing available for drawing broad conclusions about the issues and risk factors in the aftermath of natural disasters in Bangladesh is a literature review. As a result, we are doing this systematic review to learn about all the mental health issues and risk factors caused by natural disasters in Bangladesh. Bangladesh has a long history of ignoring the mental health of catastrophe survivors; this study aims to change that by shedding light on the full scope of the issues these individuals face and providing policymakers with the information they need to implement effective solutions.

## 2 Methods

### 2.1 Search strategy

Using the Preferred Reporting Items for Systematic Reviews and Meta-Analyses (PRISMA) checklist, we systematically sought out, gathered, and assessed literature on disaster-related mental health disorders (Moher et al., 2010). The PRISMA protocol was chosen after careful consideration of several methodologies and suggestions for the most effective methods of reporting systematic reviews. We used the PRISMA checklist to guide us as we extracted and tallied the data. By combining Boolean operators with Medical Subject Headings (MeSH) keywords, we were able to increase the search precision. From April to May of 2024, we used the following terms and logic: “natural disasters” OR “cyclones” OR “floods” AND “mental health” OR “mental disorders” OR “psychological distress” AND “Bangladesh.” We were able to reduce potential gaps in data collecting and guarantee complete coverage with this cross-database technique. The first step was to acquire 105 items. With an emphasis on research conducted during the recent last five years (2019–2024), studies that were eligible for inclusion looked at mental health outcomes and risk factors in the aftermath of natural disasters in Bangladesh. We quickly skimmed over all of the studies to see whether they were relevant to our research questions and aims. After the initial screening was completed, finally a total of seventeen papers were selected for data analysis.

### 2.2 Study selection criteria

A primary criterion for selecting publications was their names and abstracts. After that, the inclusion criteria were checked against the complete content of the article. Articles met the following criteria: (i) published in a peer-reviewed journal or preprint version between 2019-2024; (ii) cross-sectional surveys; (iii) conducted after a natural disaster had occurred; (iv) followed quantitative, qualitative, or mixed-method studies; (v) reported the severity and/or risk factors of mental health problems (such as anxiety, stress, or depression) in the aftermath of the disaster; and (vi) published in English. This study did not include any papers that met the following criteria: (i) conference papers, reviews, or letters; (ii) did not address the mental health consequences of natural catastrophes; and (iii) not being conducted in Bangladesh. (iv) not in a range of 2019–24 timeframe; (v) published in other language except in English.

### 2.3 Data eligibility

**Figure 1:**
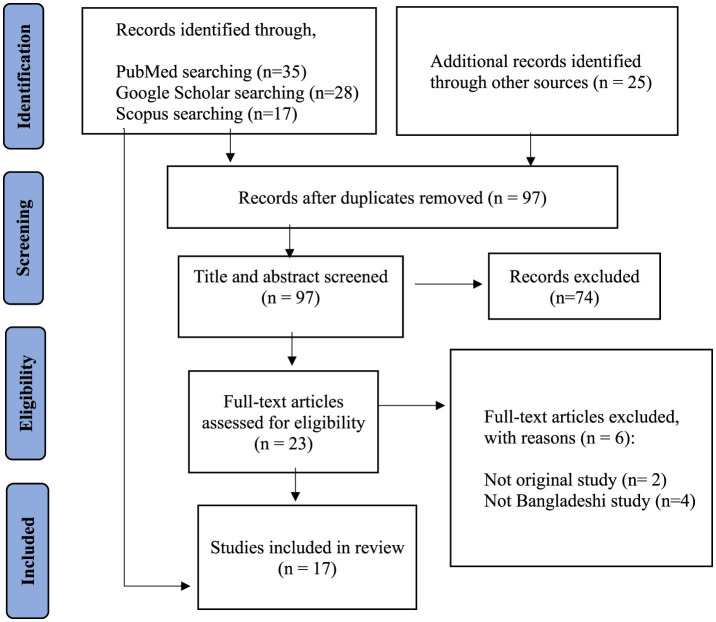
PRISMA flowchart describing the search strategy and inclusion/exclusion of studies for review (adapted from Moher et al., 2010).

There were 105 articles selected from various databases, but after removing the counterfeits, there were only 97 lefts. 74 items were discarded after going through the “*Titles and Abstracts*” of all listed articles. No less than 23 full-text articles were considered for inclusion. In the end, 17 studies that were eligible for inclusion were actually considered. Results showed that six full-text papers were not included because they were either not original studies or not from Bangladesh (see Figure 1). We have labeled each stage of the study selection process more clearly and broken down the number of studies that were included and excluded at each level. Furthermore, to make sure that readers can readily follow the review process, we have included details on inclusion and exclusion criteria directly in the flowchart. By improving alignment with PRISMA guidelines and making it easier for readers to understand, our visual presentation has considerably enhanced its overall quality (Supplementary material).

### 2.4 Data extraction

An excel file was created to organize the information procured from the studies. These following criteria were applied for extracting data-(1) author and year of publishing, (2) group and sample size (3) sampling technique, (4) sample characteristics, (5) evaluation instrument, (6) prominent risk factors of natural disaster, (7) associated risk factors.

### 2.5 Quality assessment and interpretation

We conducted a rigorous quality assessment of each included study to ensure methodological integrity, following standards established by the PRISMA guidelines (Moher et al., 2010) as well as specific evaluation tools tailored to systematic reviews. The Joanna Briggs Institute (JBI) Critical Appraisal Checklist for Systematic Reviews (Munn et al., 2020) was used for this evaluation. It examines the quality of studies according to various criteria, such as data transparency, research design, sampling adequacy, and relevance to mental health outcomes following natural disasters. To make sure that only high-quality, methodologically sound research was included in our evaluation, we evaluated each study based on parameters like bias risk, methodological consistency, clarity of findings, and ethical issues.

### 2.6 Data synthesis procedure

For quantifying study quality, we used these criteria to classify studies as either high, moderate, or poor quality and then assigned scores accordingly (Higgins and Green, 2011). To keep the validity and reliability of the review intact, we omitted studies that were considered to be of low quality (e.g., those that were highly biased or did not adhere to rigorous scientific procedures). By only including research with strong methodology in our synthesis, we were able to increase the reliability of our results and draw firm conclusions about the mental health difficulties and risk factors that have emerged in the aftermath of natural disasters in Bangladesh.

According to Popay et al. (2006), we used a structured narrative synthesis method that centered on systematic thematic analysis to find commonalities and differences in the studies and handle heterogeneity efficiently. Because of this strategy, we were able to extract and understand important findings despite variations in research methods and settings. Grouping research according to similar factors such as catastrophe type (e.g., cyclones, floods), population demographics (e.g., age, gender), and primary mental health outcomes (e.g., PTSD, anxiety, depression), we organized thematically in the synthesis of data. According to Mays et al. (2005), this allowed us to methodically identify consistent trends even when there were differences in study methods and setting.

Due to the variety in outcome measurement instruments and reporting formats among studies, it was not viable to conduct a meta-analysis on some outcome measures without compromising the integrity of individual findings. To address potential moderating factors, such as gender and socioeconomic status, that can influence mental health outcomes post-disaster, we followed best practices in systematic reviews and conducted subgroup analysis inside the narrative synthesis (Higgins and Green, 2011). Following the recommendations of the Cochrane Handbook for systematic reviews, a meta-analysis was only contemplated when there was enough consistency in the outcomes that were measured and the designs of the studies (Higgins and Green, 2011). Considering these limitations, we determined that the results would be most accurately represented by a narrative synthesis that included subgroup analyses for variables such as gender and socioeconomic position. Furthermore, in accordance with the recommendations for health outcome systematic reviews, we performed quality assessments utilizing the PRISMA checklist and AMSTAR 2 criteria (Moher et al., 2010; Shea et al., 2017) to ensure transparency.

### 2.7 Risk of bias management

To counteract possible biases, we utilized the Cochrane Risk of Bias Tool, a commonly used framework for evaluating the internal validity of randomized controlled trials (RCTs) (Corbett et al., 2014). This tool accounts for a variety of issues, including selection bias, performance bias, detection bias, attrition bias, reporting bias, and selective reporting, among others. The included randomized studies were thoroughly examined for potential validity concerns using this standardized tool, and we promptly reported them.

Sterne et al. (2016) developed the ROBINS-I (Risk Of Bias In Non-randomized Studies of Interventions) tool, which we used for non-randomized studies. This tool offers a structured approach to evaluating the potential for bias in confounding, selection, measurement, and reporting. Because a large number of the research we looked at used observational designs, we were able to use ROBINS-I to deal with the problems that come with non-randomized techniques, like bias due to non-equivalent groups or confounding variables. We were able to thoroughly examine research with different designs by using this dual-tool method and adapting our evaluation criteria accordingly (Supplementary material).

### 2.8 Ethical considerations

Recognizing the ethical factors involved in synthesizing research on mental health difficulties among people afflicted by natural disasters is vital in completing this comprehensive review. Due to the delicate nature of these populations, all initial research must adhere to strict ethical guidelines that protect participants' privacy, confidentiality, and wellbeing. Furthermore, due to the delicate nature of mental health concerns pertaining to disasters, researchers were very careful not to stigmatize victims or put them at risk of re-traumatization. By emphasizing the significance of ethical rigor in future research on mental health in disasters, this review shows respect for the victims' dignity through responsible reporting and interpretation.

## 3 Results

### 3.1 Description of the included studies

After adhering to the inclusion criteria, 17 studies conducted from 2019 to 2024 were included in the present review (see Table 1). The included studies (see Table 2) examined the impact of catastrophic natural disasters such as floods, storms, super cyclones, river bank erosion, heat waves, and earthquakes on a range of mental health outcomes, such as anxiety, depression, post-traumatic disorder syndrome (PTSD), and psychological distress.

**Table 1:** Characteristics of included studies.

**#**	**Study characteristics**	**Prevalence rate**	**Prominent mental health problems**	**Associated risk factors**	**Quality assessment score**
1.	**Study:** Fatema et al. (2023) **Major focus:** Cyclone **Study group:** Women; **Sample size:** 408; **Sampling procedure:** Systematic random sampling; **Sample characteristics:** 18 years and above **Assessment tools:** The Short Form 12 (SF-12)	Poor mental health (63%)	Poor mental health	Witnessed casualties, lost family members or relatives, food deficiency, physically injured	4
2.	**Study:** Mahmud et al. (2021) **Major focus:** Flood **Study group:** People; **Sample size:** 106; **Sampling procedure:** Stratified Random sampling; **Sample characteristics:** NR **Assessment tools:** (PCL-5), (PHQ-9), (GAD-7)	Low PTSD (5.7%), moderate PTSD (3.8%), GAD(severe)24.7%, GaD(Moderate) 49.06%, mild GAD(26.4%),	PTSD, GAD, MDD	Gender, place	3
3.	**Study:** Chandra Das et al. (2022) **Major focus:** Flood **Study group:** Adult; **Sample size:** 384; **Sampling procedure:** Convenience sampling; **Sample characteristics:** 20 years and above mean age−39.16 years **Assessment tools:** BSRS-5	A(92.19%, D(92.71%), strong irritation(44.27%) sleep disturbances (31.25%)	Anxiety, Depression, Irritation, sleep disturbances	Sex, age, monthly income, marital status	4
4.	**Study:** Hossain et al. (2021a) **Major focus:** Cyclone **Study group:** People; **Sample size:** 478; **Sampling procedure:** Cluster sampling technique; **Sample characteristics:** Mean age 37.02 **Assessment tools:** BSRS-5, HFIAS, wealth index score	Severe A (36.7%), severed (40.3%), SI(10.9%)	Anxiety, depression, suicidal ideation	Age, gender, household food insecurity, income loss	4
5.	**Study:** Hossain et al. (2021b) **Major focus:** River bank erosion **Study group:** People; **Sample size:** 611; **Sampling procedure:** Purposive sampling; **Sample characteristics:** NR **Assessment tools:** DASS-21	D (38.39%), A (76.60%), S(32.41%)	Depression, anxiety, stress	Age, sex, education, income, loss of house, loss of land, social class, social support.	3
6.	**Study:** Mamun et al. (2019) **Major focus:** Cyclone **Study group:** Women; **Sample size:** 111; **Sampling procedure:** Convenient sampling; **Sample characteristics:** aged ≥18 years **Assessment tools:** PHQ-9	D-(65%)	Depression	Age, education, physically injured, having children	3
7.	**Study:** Rahman and Gain (2020) **Major focus:** River bank erosion **Study group:** People; **Sample size:** 434; **Sampling procedure:** Purposive sampling; **Sample characteristics:** NR **Assessment tools:** Survey Questionnaire	PTSD (63.59%), MDD (26.96%),	PTSD, MDD	Vulnerable Social life, food crisis and drinking water problem, socio-economic crisis	3
8.	**Study:** Moyna et al. (2024) **Major focus:** Natural disaster **Study group:** Women; **Sample size:** 350; **Sampling procedure:** Cluster and stratified sampling; **Sample characteristics:** 18 years old or above (aged less than 60 year) **Assessment tools:** PHQ-9	D-63%	Depression	Age, having children, family income, loss of family members, loss of house & livestock,	4
9.	**Study:** Arobi et al. (2020) **Major focus:** River bank erosion **Study group:** People; **Sample size:** 100; **Sampling procedure:** Stratified sampling; **Sample characteristics:** NR **Assessment tools:** GHQ-12, cope scale	NR	Vulnerable mental health	Displacement	2
10.	**Study:** Mostafizur Rahman et al. (2023) **Major focus:** Flood **Study group:** Women; **Sample size:** 393 **Sampling procedure:** NR **Sample characteristics:** NR **Assessment tools:** DASS-21	D- 67%, A-65%, and S-37%	Depression, anxiety, stress	Age, education, housing type, social satisfaction, a place's safety rating, flood-related injury or disease, family loss, family violence,	4
11.	**Study:** Wahid et al. (2023) **Major focus:** Heatwave **Study group:** People; **Sample size:** 3606; **Sampling procedure:** Two-stage stratified sampling; **Sample characteristics:** NR **Assessment tools:** PHQ-9, GAD-7	D- 16·3% A- 6·0%	Depression, anxiety	Increased temperature, humidity, age, gender, physical disability,	4
12.	**Study:** Mamun et al. (2021) **Major focus:** Flood **Study group:** People; **Sample size:** 348; **Sampling procedure:** Simple random sampling **Sample characteristics:** NR **Assessment tools:** TSQ-10, PHQ-9, GAD-7, EHQ-6	SI- 57.5%	Suicidal ideation	Social class, less education, financial crisis	3
13.	**Study:** Tasdik Hasan et al. (2020) **Major focus:** Cyclone **Study group:** People; **Sample size:** 20; **Sampling procedure:** Purposive sampling; **Sample characteristics:** Mean age- 48.96 years **Assessment tools:** NR	NR	PTSD, SI, GAD, Depression, sleep disorder	Gender, age	2
14.	**Study:** Siddik et al. (2024) **Major focus:** Flood **Study group:** Adolescents; **Sample size:** 53; **Sampling procedure:** Purposive sampling; **Sample characteristics:** NR **Assessment tools:** PHQ-7 and PTSD-2	PTSD- Boys (60.72%), Girls (71.42%) D- Boys (84%), Girls (80%)	PTSD, Depression	Living conditions, gender.	3
15.	**Study:** Malak et al. (2020) **Major focus:** Cyclone **Study group:** Older people; **Sample size:** 24; **Sampling procedure:** Purposive sampling; **Sample characteristics:** Aged 60 and over, aged 65–69 (31.4%). **Assessment tools:** NR	NR	Vulnerable mental condition	Adverse effects on housing, agriculture, Livelihood instability, financial uncertainty, Drinking water insecurity, Physical damage	2
16.	**Study:** Nayna Schwerdtle et al. (2021) **Major focus:** Climate hazards **Study group:** People; **Sample size:** 58; **Sampling procedure:** Purposive sampling; **Sample characteristics:** (>18 years) mean age of 43 **Assessment tools:** NR	NR	Poor mental wellbeing	Social isolation, food insecurity	2
17.	**Study:** Kabir et al. (2024) **Major focus:** Sea-level rise **Study group:** People; **Sample size:** 1224; **Sampling procedure:** Purposive sampling; **Sample characteristics:** NR **Assessment tools:** K10, RLS, DASS-21, ESS	NR	Depression, anxiety, stress, psychological distress.	Environmental stressors, age, gender,	3

D, depression; A, anxiety; S-stress; PTSD, post traumatic stress disorder; GAD, generalized anxiety disorder; MDD, major depressive disorder; SI, Suicidal ideation; ESS, Environmental Stressor Scale; DASS, 21, depression, anxiety, stress scale; RLS, Resource Loss Scale; K10, Kessler Psychological Distress Scale;PTSD, 2, posttraumatic stress disorder scale; PHQ, 7, patient health questionnaire scale; TSQ, 10, Trauma Screening Questionnaire (TSQ, 10), GAD, 7 generalized anxiety disorder scale, EHQ, 6, Economic Hardship Questionnaire; GHQ, 12, General health questionnaire scale; BSRS, 5, brief symptom rating scale, HFIAS, Household Food Insecurity Access Scale; PCL, 5, Posttraumatic Stress Checklist; NR, Not Reported.

**Quality Assessment Score:** Scores range from 1 to 5, with 1 indicating poor quality, score 2: fair quality, score 3: moderate quality, score 4: good quality and 5 indicating excellent quality based on criteria such the rigor of the study design, the sample size, the methods used, and how well the study addressed potential biases.

**Table 2:** Summary of key findings of included studies.

**#**	**Study Details**	**Summary of research findings**
1.	**Title:** Physical and mental health status of women in disaster affected areas in Bangladesh; **Author:** Fatema et al. (2023) **Research design:** Quantitative **Method:** Survey method	This study sought to investigate both physical and mental wellbeing status as well as related risk factors of women living in Bangladeshi areas that are frequently affected by disasters. Poor physical and mental health is indicated by the fact that 65.9% and 63.0% of all participants, respectively, rated lower than the established cut-off points on the PCS-12 and MCS-12 subscales. The results demonstrated that people's physical and mental health suffered considerably more when they witnessed fatalities, lost loved ones, were forced to seek refuge, or had to go without food during emergencies. Furthermore, individuals who experienced physical harm and/or sought medical attention from a practitioner during or following the crisis were shown to have lower mental health scores.
2.	**Title:** Geographic variability of post-disaster mental health: Case study after the 2017 flood in Bangladesh **Author:** Mahmud et al. (2021) **Method:** Case study covering both quantitative & qualitative	Researchers found that individuals experience low to moderate levels of post-traumatic stress disorder (PTSD), generalized anxiety disorder (GAD), and major depressive disorder (MDD) following a flood. The prevalence of mental disorders changed in terms of gender. Women showed higher levels of PTSD, GAD and MDD. Environmental setting plays a vital role in developing PTSD, GAD and MDD. People who live in homogenous environmental settings near the river may experience high rates of PTSD; on the other hand, low levels of PTSD are found significant when they live at a considerable distance area from the river. It is also found that some heterogeneous geographical settings can change the pattern of PTSD.
3.	**Title:** Mental Health Symptoms Among Flood Victims in Madaripur District in Bangladesh: A Cross-sectional Study **Author:** Chandra Das et al. (2022) **Method:** Cross sectional study	This study investigated the effects of floods on mental health and investigated the major risk factors for mental illness associated with flooding in the Bangladeshi region affected by flooding. Anxiety levels among the individuals range from 17% to 40%, with mild to severe symptoms. Additionally, our study revealed that 18% to 44% of participants had mild to severe depression. In comparison to women, who only acquired serious mental health symptoms in 20.49% of cases, males experienced severe mental health symptoms at a rate of 31.03%. Of the individuals in the age range of 20 to over 60, 45.05 per cent who were between the ages of 41 and 50 experienced significant mental health signs. Evidence has demonstrated that study participants with higher numbers of cohabiting individuals, more kids, fewer individuals as their main source of earnings, a lower level of schooling, and additional sources of income were considerably more likely to experience psychological warning signs.
4.	Title: Household food insecurity, income loss, and symptoms of psychological distress among adults following the Cyclone Amphan in coastal Bangladesh **Author:** Hossain et al. (2021a) **Method:** Cross sectional study	After Cyclone Amphan, moderate-to-severe mental health problems are observed to be highly prevalent in coastal areas of Bangladesh. After the cyclone, moderate-to-severe level of mental health syndromes were recorded by 66% of the adults, while severe food insecurity was reported by 40.8% of the adults. The findings show a direct correlation between moderate-to-major mental health symptoms and major household food insufficiency. It suggests that having a stable food supply at home significantly lowers psychological stress related to suicidal thoughts. Stressors from the aftermath of the cyclone, such as lost wages and poor housing, were also significant factors in suicidal thoughts. 36.7% of participants seemed to have experienced severe anxiety, and 40.3% of people appeared to have depression
5.	**Title:** Effects of riverbank erosion on mental health of the affected people in Bangladesh **Author:** Hossain et al. (2021b) **Method:** Cross-sectional household survey	The main goal is to investigate how Bangladeshi riverbank erosion affects mental health issues like stress, anxiety, and depression. There are two groups in this study: one that was exposed to river bank erosion and another that was not. Depression, anxiety, and stress (DAS) were found in 38.30%, 76.60%, and 32.41% of the exposed group, respectively. These rates were immensely greater than those of the non-exposed group. Compared to the male participants, the female individuals had higher rates of depression, anxiety, and stress. Individuals who have three or more children, a lower monthly income, and no land for cultivation are at a higher chance of developing stress, anxiety, and depression. In terms of occupation, homemakers had the highest rate of depression. There were considerably greater rates of stress (54.59%), anxiety (95.20%), and depression (63.32%) among those who relocated.
6.	**Title:** Prevalence of depression among Bangladeshi village women subsequent to a natural disaster: A pilot study **Author:** Mamun et al. (2019) **Method:** Cross-sectional survey	Women who live in disaster-prone locations are particularly susceptible to experience mental health issues including depression.64.9% of the ladies had depression. In addition to a variety of other post-disaster repercussions, 36.0% of respondents reported bodily injuries, 27.9% missed work and so lost money, and 17.1% reported the demise of family members. Individuals aged 18 to 30, having a source of income, physical injuries sustained during natural disaster, and after-disaster work absenteeism were found to be the associated with depressive symptoms. Women having younger age appeared to be especially affected by depression. The lowest incidence of depressive symptoms was seen among women who were able to depend on their partners and other loved ones for economic and non-financial assistance.
7.	**Title:** Adaptation to river bank erosion induced displacement in Koyra Upazila of Bangladesh **Author:** Rahman and Gain (2020) **Method:** Mixed (both quantitative & qualitative)	People's life is significantly affected by river bank erosion, which progressively affects their social, psychological, and economic hardships. It was discovered that 26.27% of participants live next to highways and barrages because of erosion, and around 82.49% of respondents' houses were destroyed. Because of river bank erosion, over 85.02% of participants were domestically relocated or lost their houses. A significant proportion of the respondents—roughly 76.27%—had their employment status severely impacted; 48.39% had to change their source of income and began fishing; 28.34% of households are experiencing a severe food crisis; 36.18% of respondents have their income declining; 32.02% have lost cultivable land; and 91.94% have psychological stressors like anxiety, depressive symptoms, insomnia problems, major depressive disorder, PTSD, and somatoform disorder as a result of river bank erosion.
8.	**Title:** Depressive symptoms among women in disaster-prone region in Bangladesh **Author:** Moyna et al. (2024) **Method:** Cross-sectional survey	Women are among the most important victims of disasters in terms of mental health. It was discovered that 63% of the subjects suffered from moderate to severe depression. Similarly, it was found that major hazardous things for women in terms of developing depression in Bangladesh, a country vulnerable to natural disasters, included being married, having kids, having to deal with physical harm, losing one's job due to a disaster, having damage to one's home or crops, family disputes, losing household livestock, and worrying about prospective losses in the years to come. This is because, when compared to men, prevalence of assault based on gender is greater among women., economic instability, caregiving responsibilities, and unstable social support during disasters. Approximately 78.3% of those participants expressed concern about going through a natural disaster again.
9.	Title: Impact of River Bank Erosion on Mental Health and Coping Capacity in Bangladesh **Author:** Arobi et al. (2020) **Method:** Cross-sectional study	This study compared the coping strategies and mental health of Bangladeshi residents impacted by river bank erosion and to those who were not affected. Displaced populations following river bank erosion face stressors associated with social network disruption, familial separation, and social isolation. Overall, the study discovered that people's mental health was negatively impacted by river bank erosion. It was discovered that the effect of natural catastrophes over the affected population's mental health repercussions varies. It implies that psychological resilience improves one's capacity for recovery and aids in adjusting to setbacks while witnessing natural disasters.
10.	**Title:** Impact of Disaster on mental health of women: a case study on 2022 flash flood in Bangladesh **Author:** Mostafizur Rahman et al. (2023) **Method:** Cross-sectional survey	It examined women's flood-related mental health issues. In terms of affecting depression, anxiety, and stress, roughly 67%, 65%, and 37% of women reported experiencing each. Furthermore, after suffering family violence during a flood, 89%, 88%, and 58% of female indicated having major or immensely major depression, anxiety, and stress. Age, education, kind of housing, social satisfaction, safety rating of a location, illness or injury related to flooding, family violence, loss of a family member, and wealth damage following 2022 flood were all linked to depression. With the exception of schooling and the area's current flood safety rating, every variable linked to depression was associated with stress. Older women living in poor infrastructure, those who saw the demise of family members, experienced family violence and property devastation as a result of the flood, all reported psychological health issues.
11.	**Title:** Climate-related shocks and other stressors associated with depression and anxiety in Bangladesh: a nationally representative panel study **Author:** Wahid et al. (2023) **Method:** Panel survey	This investigation examined the sociodemographic and climate-related determinants of anxiety and depressive disorders using information obtained from a nationwide representative survey conducted in Bangladesh. There exists a high correlation among the development of depression and anxiety with natural disasters and other stressors in Bangladesh. The study reported a prevalence of 16.3% for depression, 6.0% for anxiety, and 4.8% for co-occurring depression and anxiety. Increased temperature was linked to anxiety, but not depression. Individuals with physical disabilities reported much greater rates of depression and anxiety than those without disabilities. Biological sexual identity had a notable association with depression, with females experiencing greater levels than male.
12.	**Title:** Suicidal Behavior and Flood Effects in Bangladesh: A Two-Site Interview Study **Author:** Mamun et al. (2021) **Method:** A cross-sectional survey study	Socio-demographic factors and psychological disorders following flood are linked to suicidal behaviors in Bangladesh. According to the study, 57.5% people who survived from flood had suicidal ideation, while 5.7% planned suicide and 2.0% attempted suicide. Suicidal thoughts and actions are associated with a number of factors, including coming from a lower socioeconomic background, having family members who have a lower educational attainment, having been severely impacted by the flooding, experiencing PTSD, despair, and feelings of financial jeopardy.
13.	**Title:** Exploring mental health needs and services among affected population in a cyclone affected area in costal Bangladesh: a qualitative case study **Author:** Tasdik Hasan et al. (2020) **Method:** Qualitative case study	This study got those cyclones had a variety of psychological and social effects on the people, such as severe stress disorder, sleep disturbance, PTSD, GAD, suicidal ideation, and depression. Survivors of disasters expressed hopelessness, frustration, grief, and guilt following such a devastating disaster. It is also stated that the natural disaster warning system was viewed as an adjuvant thing for instilling worry and fear among the local illiterate population. Adolescents' behavior pattern was changed due to experiencing natural disasters. It had a significant impact on family connections and psychosocial aspects. Divorces, domestic violence incidents, and current suicide attempts occurred in the aftermath of such calamities.
14.	**Title:** Climate change, natural disasters, and mental health of adolescents: A qualitative study from Bangladesh **Author:** Siddik et al. (2024) Method: qualitative study	Floods have severely interrupted the daily routines of individuals impacted, causing a notable alteration in their habits, particularly among youths. The flood's severe economic consequences, as well as the increased mental stress it caused, exacerbated the situation. Our findings revealed that a sizable number of boys and girls developed PTSD and despair. Specifically, 60.72% of boys and 71.42% of girls had PTSD, whereas 80% of girls and 84% of boys were depressed. PTSD, sadness, and psychological distress appear to be prevalent among adolescents who have been forced to live in severe conditions in flood shelters. Teenagers' behavior grew irregular. They had incredibly short tempers when they were angry. The gender disparity was noticeable here, with the boys.
15.	**Title**: “We are feeling older than our age”: Vulnerability and adaptive strategies of aging people to cyclones in coastal Bangladesh **Author:** Malak et al. (2020) **Method:** Qualitative study	The results of this research demonstrate that elderly people of the society are more susceptible to cyclones, just like children are. When a cyclone makes landfall, older people are particularly vulnerable. Furthermore, they are also vulnerable due to their deteriorating mental faculties and stamina. Vulnerable condition is at highest levels when appropriate key measures are lacking, such as societal consciousness, education, and restricted availability of medical services. Older people are often denied access to basic necessities like food, familial support, and sanitary services, which leaves them vulnerable both mentally and physically. This study also discovered that, senior citizens still had problems with the water they consumed. When it comes to receiving and reacting to information from early alert systems of natural disasters, they seemed in fragile condition.
16.	**Title:** A Risk Exchange: Health and Mobility in the Context of Climate and Environmental Change in Bangladesh—A Qualitative Study **Author:** Nayna Schwerdtle et al. (2021) **Method:** Qualitative study	Floods, hurricanes, and cyclones caused individuals to be injured, drown, or endure psychological anguish. Migrants described how their physical and mental health changed over time in connection to their hardships following natural disaster. Migrants reported a considerable reduction in food security, citing shortages, poor quality, and safety. Key problems included inconsistent availability of water, poor drinking water quality, and the need for boiling water in a setting limited and expensive power. Individuals who described their mental condition and wellbeing in the context of a lack of serenity and devastating loss. Several participants gently indicated concerns about their poor mental health and wellbeing, citing a lack of peace depending on sense of social and cultural loss. This study discovered that climate-related migration can disrupt social bonds, potentially having adverse consequences for mental health and wellbeing.
17.	**Title:** Sea-level rise and mental health among coastal communities: A quantitative survey and conditional process analysis **Author:** Kabir et al. (2024) **Method:** Quantitative study	This study conducted to determine the influence of sea level rise emerged from environmental stressors over mental health. It has been established that individuals living in environmentally fragile Coastal areas are more vulnerable to psychological discomfort, depression, anxiety, and stress. Along with that, educational background and income condition were discovered to influence psychological health consequences, simultaneously, marital status had same effect. People who got divorced reported higher rates of mental anguish, anxiety, and stress than unmarried people. Compared to those without schooling, individuals with some educational background reported feeling less anxious, nervous, and depressed. Family who got higher levels of income showed lower rates of mental discomfort, anxiety and depression. Aged people and female are reported to be highly sensitive to environmental stressors. Cultural and cultural constraints, like limited access to education and wealth, may worsen women's vulnerabilities. Mental health outcomes varied across religious affiliations.

Out of the 17 papers that were considered, nine employed cross-sectional surveys, four utilized qualitative methods, two applied quantitative methods, and two used mixed methods. Furthermore, 10 papers about floods and cyclones, three studies concerning river bank erosion, two studies regarding heatwaves and sea level rise, and two studies about general natural catastrophes were included in the research.

### 3.2 Prominent mental health problems following natural disaster

Natural disaster has an immense impact on people's mental health. Mental health disorder like depression, anxiety, stress, and somatoform disorder plays a dominant role in people's mental wellbeing. Nevertheless, the following discussion will include the specific prevalence rates of mental health issues as reported in the included research.

#### 3.2.1 Depression

**Figure 2:**
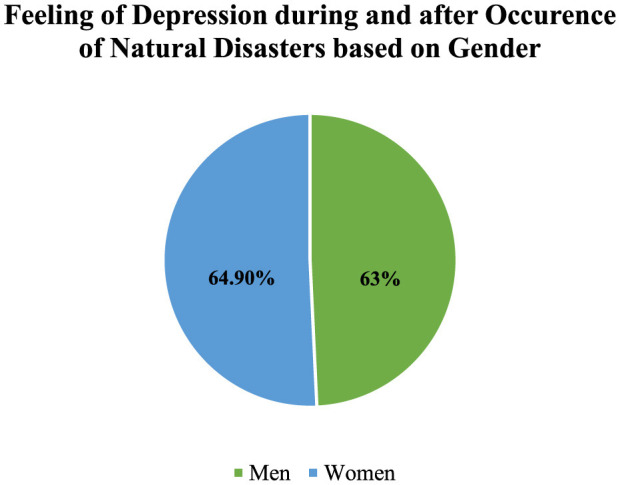
Prevalence of depression among sufferers based on Moyna et al. (2024), Mamun et al. (2019), Rahaman et al. (2023) findings.

People after natural disasters are more likely to suffer from depression. A total of eight research found that natural catastrophes can cause depression in humans, with percentages ranging from 16.3% to 92.71% (Wahid et al., 2023; Hossain et al., 2021b,a; Chandra Das et al., 2022). The percentage of flood victims who suffered from moderate to severe depression ranged from 18% to 44% (Chandra Das et al., 2022). Depression was reported by 63%, 64.9%, and 67% of women in studies including men and women, respectively (Moyna et al., 2024; Mamun et al., 2019; Mostafizur Rahman et al., 2023). A study found that cyclones have multiple psychological consequences on people, one of which was depression (Tasdik Hasan et al., 2020). Based on gender identification considering depression which was shown in Figure 2.

#### 3.2.2 Stress and anxiety

Anxiety is a major element brought on by natural disasters, according to five research. Natural disasters such as heatwaves, cyclones, floods, and river bank erosion might cause anxiety in 6%, 36.7%, 76.60%, and 92.19% of the people surveyed (Wahid et al., 2023; Hossain et al., 2021a,b; Chandra Das et al., 2022). Research including female participants indicated that 88% of them experienced anxiety (Mostafizur Rahman et al., 2023). Anxiety levels range from 17 to 40% and symptoms from mild to severe, according to a study (Chandra Das et al., 2022). Furthermore, stress is common after natural disasters, according to three studies. A concerning aspect of river bank erosion, according to 32.41% of respondents surveyed (Hossain et al., 2021b), is stress. One of the many psychosocial impacts of cyclones on humans was the development of acute stress disorder, according to research (Tasdik Hasan et al., 2020). In addition, research on women in coastal regions revealed that 58% had severe stress (Mostafizur Rahman et al., 2023).

#### 3.2.3 Major mental health disorder (GAD, PTSD, suicidal ideation, and sleep disorder)

Individuals develop a mild to moderate level of major depressive disorder following a flood. (Mahmud et al., 2021). A study based on women revealed that 91.94% of respondent's experience major depressive disorder as a result of river bank erosion (Rahman and Gain, 2020).

**Figure 3:**
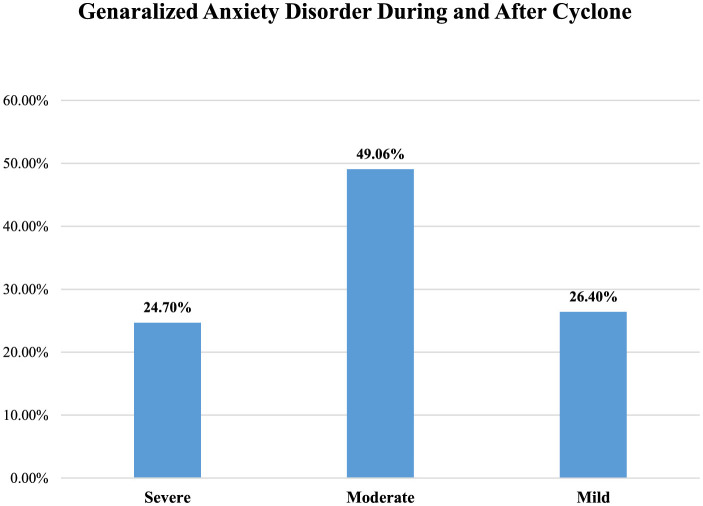
Prevalence of GAD among sufferers of cyclone based on Mahmud et al. (2021), Wahid et al. (2023) findings.

A study conducted on cyclone-affected people found that generalized anxiety disorders are one of the devastating factors that impact grievously to people (Tasdik Hasan et al., 2020). Having experienced the flood, people reported low to moderate levels of generalized anxiety disorder (Mahmud et al., 2021). Moreover, research revealed that 24.7% population reported a severe level of generalized anxiety disorder, 49.06%population reported a moderate level of generalized anxiety disorder, and 26.4% population reported a mild level of generalized anxiety disorder after the onset and end of the cyclone (Wahid et al., 2023) (see Figure 3).

In a study that examined the effects of floods, the frequency of post-traumatic stress disorder was found to be low to moderate among the participants (Mahmud et al., 2021). 5.7% population reported low level of post-traumatic stress disorder, 3.8%population reported moderate level of post-traumatic stress disorder (Mahmud et al., 2021). According to Siddik et al. (2024), 60.72% of boys and 71.42% of girls reported PTSD following floods. The rest of the study showed cyclone had a severe effect over population that caused PTSD (Tasdik Hasan et al., 2020).

**Figure 4:**
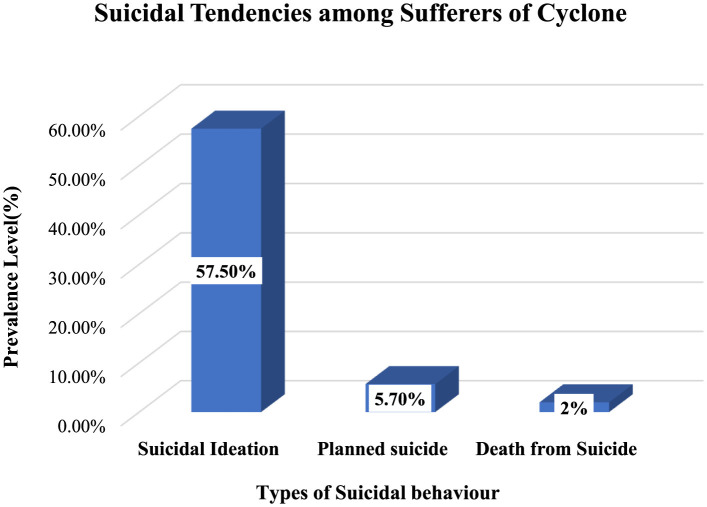
Prevalence of Suicidal tendencies among sufferers of cyclone based on Mahmud et al. (2021) findings.

Prevalence of suicidal ideation following natural disaster found in three studies. Studies conducted over cyclone affected people which showed suicidal ideation is prominent factor of natural disasters (Tasdik Hasan et al., 2020) and 10.9% people got suicidal ideation (Hossain et al., 2021a). Along with that, Mamun et al. (2021), reported that 57.5% of flood affected people had suicidal ideation, while 5.7% planned to suicide and 2.0% endeavored to do suicide (see Figure 4).

Three studies predicted sleep disturbances as dominant risk factor following natural disaster. A study depicted that 31.25% people reported sleep disturbances due to having experienced flood (Chandra Das et al., 2022). The role of natural disaster over sleep disturbances found as significant factor (Tasdik Hasan et al., 2020). Like the two studies, Rahman and Gain (2020), showed that 91.94% of respondents have insomnia following river erosion.

### 3.3 Risk factors associated with prominent mental health problems

The risk factors for mental health problems are categorized into four distinct groups– (i) Demographic factors, (ii) Socio-economic factors (iii) Behavior and health related factors, and (iv) other factors. However, details about the mental health risk factors are discussed below.

#### 3.3.1 Demographic factors

##### 3.3.1.1 Age

A study depicted age as a major risk factor to psychological problems (Mostafizur Rahman et al., 2023). Individuals between the age range of 41 and 50 suffered major mental health issues (Chandra Das et al., 2022). Women in a younger age group proved to be more impacted by depression; the elderly age group had the second highest rate of depression (Mamun et al., 2019; Kabir et al., 2024; Malak et al., 2020). PTSD, depression, and psychological distress following flood appear to be prevalent among teenagers (Siddik et al., 2024).

##### 3.3.1.2 Gender

Gender plays a significant role here. Relationship between gender and mental disorder following natural disasters was found in five studies, where one study showed that women got higher levels of PTSD, GAD and MDD (Mahmud et al., 2021). Similarly it is claimed that environmental stressors tend to affect women more sensitively (Kabir et al., 2024), along with that Wahid et al. (2023) depicted that woman reported higher levels of depression. For instance, women are vulnerable to natural disaster following which 80% girls faced depression due to witnessing natural disasters (Wahid et al., 2023). But, the peculiar finding, that is, males were found to higher levels of mental health symptoms maintaining a rate of 31.03% (Chandra Das et al., 2022).

##### 3.3.1.3 Living location

In a study, it was found that around 82.49% of respondents' houses were completely demolished due to erosion who used to live adjacent to embankments (Rahman and Gain, 2020). Residing in places prone to disasters was identified as a substantial risk factor for mental health issues. Research indicates that women living in disaster-prone areas are more prone to experiencing mental health issues, such as depression (Mamun et al., 2019). Simultaneously, developing level of PSTD following flood depends on the distance of your living location from river (Mahmud et al., 2021).

##### 3.3.1.4 Living infrastructure

Women living in poor infrastructure, having property damage from the flood, all cited mental health difficulties (Mostafizur Rahman et al., 2023). A study revealed that adolescents who have been forced to reside in impoverished conditions in flood shelters are more susceptible to suffer from post-traumatic stress disorder (PTSD), depression, and psychological discomfort. (Siddik et al., 2024). Simultaneously, one study showed that poor housing, played as significant factors in developing suicidal thoughts (Hossain et al., 2021a).

##### 3.3.1.5 Marital status

Marital status had an association with mental disorder symptoms. Moyna et al. (2024) reported that marital status is significantly correlated with depression. Whereas, it was showed that divorced people rated higher levels of anxiety, anguish and stress than unmarried people (Kabir et al., 2024).

##### 3.3.1.6 Number of children

The relationship between a number of children and mental health problems is showed significant in two studies. The prevalence of mental health problems was found to be significantly higher among participants having more children (Chandra Das et al., 2022). People who have three or more children have more chances of developing stress, anxiety, and depression (Hossain et al., 2021b).

##### 3.3.1.7 Lower education

The magnitude of mental health disruption was depicted to be related to education. Participants having lower levels of education showed higher levels of mental health symptoms (Chandra Das et al., 2022); similarly, it was also reported that spending more time in education is strongly related to lower levels of discomfort, anxiety, and depression (Wahid et al., 2023; Mostafizur Rahman et al., 2023; Kabir et al., 2024). Another study conducted by Mamun et al. (2021) found that having less education is associated with developing suicidal behavior.

##### 3.3.1.8 Family conflicts and loss of dear ones

Having experienced natural disasters, people reported family conflicts as a notable risk factor that provokes to development of mental health problems. Women witnessed higher levels of gender violence (Moyna et al., 2024). 89%, 88%, and 58% of females who had been victims of family feuds during a flood had serious or very severe depression, anxiety, and stress (Mostafizur Rahman et al., 2023). It had an immense impact on the psychosocial aspect of individuals following which divorce and suicide attempts occurred (Tasdik Hasan et al., 2020). Two studies reported that the demise of family members as the aftermath of natural calamities played a significant risk factor in developing mental disorders; 7.1% of participants reported the demise of their family members due to natural disasters (Mamun et al., 2019). Losing a family member due to a flash flood was related to severe depression (Mostafizur Rahman et al., 2023; Al-Mamun et al., 2023).

##### 3.3.1.9 Land for cultivation

Mental health sufferings may increase due to having less cultivatable land. The river bank erosion has resulted in the loss of land for 32.02% of the participants (Rahman and Gain, 2020). Individuals having no land for cultivation reported their higher rate of stress, anxiety, and depression (Hossain et al., 2021b).

##### 3.3.1.10 Loss of domestic animals

Losing domestic animals following natural disasters was found as predictive factor related with mental health problems. Due to devastating domestic loss, participants reported their vulnerable mental health and wellbeing (Nayna Schwerdtle et al., 2021; Al-Mamun et al., 2024). Another study depicted that depression is correlated with domestic animal loss- losing domestic animals defined as major risk factor for depression (Moyna et al., 2024).

#### 3.3.2 Socio-economic factors

##### 3.3.2.1 Environmental settings

Environmental stressors cause families and vulnerable individual's great distress. environmental settings play significant role developing mental disorders. PTSD was more common among those who had previously lived in somewhat isolated riverside communities (Mahmud et al., 2021). The pattern of PTSD could be changed following heterogeneous geographic settings (Moyna et al., 2024; Rahaman et al., 2023).

##### 3.3.2.2 Social support

Social support is a fundamental thing that has a reverse relationship with developing mental health problems during natural disasters. Vulnerability is currently at its highest level due to a lack of sufficient necessary activities, including social consciousness, instruction, and limited access to healthcare resources (Malak et al., 2020). Individual experienced higher rates of mental health symptoms based on unstable social support during natural disaster (Mostafizur Rahman et al., 2023; Al-Mamun et al., 2024).

##### 3.3.2.3 Disaster warning system

Two studies reported that disaster warning systems played a risk factor which provoked to emerge of mental health disorders. People showed extreme vulnerability in response to receiving and responding to early warning system messages of natural catastrophes (Malak et al., 2020). The sounds of natural disasters, like alarms, thunder, lightning, and loud horns made to aware people could induce panic and anxiety among survivors of natural disasters (Tasdik Hasan et al., 2020).

##### 3.3.2.4 Displacement

Displacement due to natural disasters is the most vital significant factor to induce mental health problems. Few studies reported that due to river erosion, more than 85.02% of respondents were displaced, relocated, or became homeless (Rahman and Gain, 2020). Participants reported higher rates of stress (54.59%), anxiety (95.20%), and depression (63.32%) following their displacement of living places because of natural disasters (Hossain et al., 2021b). Migrants highlighted the transformation of their physical and mental well-being over time due to their experience of being displaced (Nayna Schwerdtle et al., 2021). Displaced populations due to river bank erosion experience pressures such as social network difficulties, familial separation, and social isolation (Arobi et al., 2020).

##### 3.3.2.5 Food crisis

Food crisis is strongly correlated with mental health problems. After the cyclone, 40.8% of adults faced severe food shortages and 66.0% had moderate to severe mental health problems (Hossain et al., 2021a). Similarly, 28.34% of households face a severe food crisis (Rahman and Gain, 2020). A study found that having a consistent food supply at home after natural disasters greatly reduces psychological stress associated with suicidal ideation (Rahman and Gain, 2020).

##### 3.3.2.6 Financial support

Two studies described financial crisis is notable risk factor in developing mental health problems following disaster. Mamun et al. (2021) elucidated that suicidal conduct is associated with feelings of financial threat and economic struggle. whereas, the prevalence of depressive symptoms was lowest among women who were able to get economic and other forms of assistance from their partners and other family members (Mamun et al., 2019; Parvez et al., 2023).

##### 3.3.2.7 Income loss

Another crucial risk factor related mental health disorders is income loss. The frequency of mental health problems was found to be significantly greater among peoples who had fewer primary source of income or who experienced income loss following natural disaster (Chandra Das et al., 2022). Stress, anxiety, and depression are more prevalent among individuals with lower monthly incomes (Hossain et al., 2021b). Decreased wages was a notable factor developing suicidal behavior, Suicidal behavior is associated with being from a lower-income family (Hossain et al., 2021a; Mamun et al., 2021). 27.9% participants missed work and therefore reported lost money after natural disaster (Mamun et al., 2019), 36.18% of respondents have shown their income declining (Rahman and Gain, 2020).

##### 3.3.2.8 Vulnerable livelihoods

River bank erosion has a substantial impact on the livelihoods of individuals, which in turn leads to gradual social, psychological, and economic difficulties (Rahman and Gain, 2020). Depression was associated with the safety rating of a living location, the condition of their housing, and the devastation to their property caused by the 2022 flash flood (Mostafizur Rahman et al., 2023).

##### 3.3.2.9 Living conditions

People lost their cattle, arable land, and sometimes their homes as a result of natural calamities. After that, many impacted either lost their homes or were forced to relocate (Rahman and Gain, 2020). Consequently, people are forced to stay in shelters, which pose security and privacy risks (Siddik et al., 2024). As a result of river bank erosion, displaced communities face multiple challenges, including issues with social networks, separation of families, and social isolation (Arobi et al., 2020). Natural disasters frequently leave people with acute food crises and water contamination (Hossain et al., 2021a). Additionally, a financial crisis emerged as a result of diminished income and the loss of revenue source for those impacted, making their living conditions more challenging (Chandra Das et al., 2022; Mamun et al., 2021). In a broader sense, people in Bangladesh who are badly impacted by natural disasters have a very difficult time of it, which can lead to mental health issues like depression, anxiety, and thoughts of suicide (Hossain et al., 2021b; Rahman and Gain, 2020).

#### 3.3.3 Behavior and health related factors

##### 3.3.3.1 Behavior pattern

Two studies found that individual's behavior pattern could be changed following natural disaster. Siddik et al. (2024) reported that Teenagers' behavior grew irregular. They had incredibly short tempers when they were angry during natural disaster. Boys showed higher rates in changing their behavior. Whereas, a study predicted that adolescent's behavior pattern was changed significantly due to experiencing natural disasters (Tasdik Hasan et al., 2020).

##### 3.3.3.2 Physical injury

A study done by Mamun et al. (2019) depicted that 36% participants reported physical injury following natural disasters. Physical injuries induced by natural disasters was identified as major risk factor of depression in Bangladesh (Moyna et al., 2024; Mostafizur Rahman et al., 2023). Having physical injuries in the age range of 18 to 30 years were related to depressive symptoms (Mamun et al., 2019).

##### 3.3.3.3 Physical disability

The presence of a physical disability may elevate the likelihood of developing mental health issues during a natural disaster. Individuals with physical disabilities reported much greater rates of depression and anxiety than those without disabilities (Wahid et al., 2023).

##### 3.3.3.4 Risk of resilience

Natural disasters inflict a devastating impact on the residents of Char (island) in Bangladesh because of their low resilience. Their minimal resilience is the consequence of their isolation and lack of resources (food, money, healthcare, etc.) due to natural disasters (Sarker et al., 2020). People who faced severe natural disasters like river bank erosion showed low resilience (Arobi et al., 2020). On the contrary, building community resilience after a natural disaster can diminish the psychological impact of natural disasters in Bangladesh (Uddin et al., 2020). It was found that to enhance their resilience to disasters, communities must mitigate risks and address resource disparities, involve local populations in mitigation efforts, establish organizational connections, enhance and safeguard social support systems, and prepare for contingencies, necessitating adaptability (Norris et al., 2008).

#### 3.3.4 Other factors

Increased temperature was linked to anxiety, but not depression (Wahid et al., 2023). Depression and post-traumatic stress disorder (PTSD) were more common in females than in males (*p* > 0.001), although there were no significant differences in the prevalence of these disorders according to age or marital status. In addition, staying in joint families and experiencing the loss of a house were associated with higher rates of PTSD and sadness (*p* < 0.01). Individuals who smoked more cigarettes after entering the camps were much more likely to suffer from depression (*p* < 0.01) (Mubeen et al., 2013). Isolation is a common practice among illiterate women in contemporary culture, which contributes to their psychological isolation and the development of mental health problems. The study revealed a significantly higher occurrence of mental health disorders among those who dwelt with a greater number of cohabitants (Rahman and Gain, 2020; Mubeen et al., 2013). Developing suicidal behavior is associated with being from a lower-class family (Mamun et al., 2021; Kalam and Al-Mamun, 2024). Besides, 91.94% of respondents have psychological stressors like anxiety, depression, somatoform disorder as a result of river erosion (Rahman and Gain, 2020).

## 4 Discussion

Natural catastrophes and mental health continue to be major concerns in contemporary public health. Air, soil, and water pollution are just a few of the exposures that have been linked to negative effects on physical and mental health, and even early mortality. Natural disasters including tornadoes, floods, erosion, wildfires, and droughts can cause significant problems in the long run. In order to better comprehend the most common mental health issues caused by natural catastrophes, this systematic review set out to do just that. In the aftermath of devastating natural catastrophes, this comprehensive review found that few serious mental health issues surfaced (see Table 3).

**Table 3:** Prevalence of mental health problems and associated risks factors among sufferers of natural disasters (most acute types are listed below).

**Serial no**.	**Mental health problems**	**Existing scenario**	**Mostly affected**
01	Depression	- About 63% men - 64.9% women (Moyna et al., 2024; Mamun et al., 2019; Mostafizur Rahman et al., 2023)	Women>Men
02	Post-Traumatic Stress Disorder	- About 5.7% low PTSD - 3.8% moderate PTSD (Mahmud et al., 2021)	Independent in most cases
03	Post Traumatic Sleep Disorder	- About 60.72% boys - 71.42% girls (Siddik et al., 2024; Rahaman et al., 2023)	Girls> Boys
04	Suicide	- About 57.5%- suicidal ideation - 5.7%- planned suicide - 2% death from suicide (Mahmud et al., 2021)	Women mostly but men also fell prey to it.
05	Generalized Anxiety Disorder (GAD)	- About 24.7% severe GAD - 49.06% moderate - 26.4% mild (Mahmud et al., 2021; Tasdik Hasan et al., 2020)	Mostly female but also prevails among male
**Associated risks factors**			
01	Age	-Younger women -The elderly group -Teenagers mostly suffer from PTSD, depression and psychological depression. (Mamun et al., 2019)	Younger women>elderly>teenagers
02	Gender	-Women mostly suffer. -About 80% women suffer due to natural disasters. (Wahid et al., 2023)	Women> men
03	Living location	−82.49% lost their residences during natural disaster (Mostafizur Rahman et al., 2023)	Coastal area>urban area
04	Living location (shelter house)	- Property loss induced mental difficulties (Mostafizur Rahman et al., 2023) - Living in congested shelter homes mostly invite PTSD, depression and psychological distress among sufferers (Siddik et al., 2024)	Independent
05	Marital status	- Divorced people mostly suffer more than unmarried (Mubeen et al., 2013).	Divorced people>unmarried people
06	Number of children	- Family owns more children suffer in the long run (Chandra Das et al., 2022) - More children in family welcomed stress, anxiety and depression among family members (Hossain et al., 2021b)	Extended family>nuclear family Family with more children> family with less children
07	Gender violence and educational qualification	- Revealed that gender violence owing to the onset of flood fluctuates mental health conditions. - 89% = severe depression - 88%= anxiety - 55%= stress (Mostafizur Rahman et al., 2023)	Mostly women

Natural disaster has an immense impact over people's mental health. It causes people's poor physical and mental health (Fatema et al., 2023). Analyzing those included studies, depression, anxiety, stress, suicidal ideation, post-traumatic stress disorder, major depressive disorder, generalized anxiety disorder and sleep disturbances are found as major mental health problems. Prevalence of depression, anxiety and stress, suicidal ideation range from 16.3% to 92.71%, 6% to 92.19%, 32.41% to 58%, 10.9% to 57.5% among people as consequences of natural disasters like heatwave, cyclone, flood, river bank erosion (Wahid et al., 2023; Chandra Das et al., 2022; Hossain et al., 2021a,b; Mostafizur Rahman et al., 2023; Mamun et al., 2021). People having generalized anxiety disorder following natural disaster showed mild to severe level gad (Mahmud et al., 2021). A study depicted that 91.94% of respondents have psychological stressors like insomnia, major depressive disorder, post-traumatic stress disorder as a result of natural disaster (Rahman and Gain, 2020). Magnitude of mental health problems may vary in terms of natural disasters types, measuring tool etc. For instance, depression in found mostly among flood and cyclone affected people (Chandra Das et al., 2022; Hossain et al., 2021a). However, most of the studies predicted those factors significantly as leading mental health problems following natural disaster. Therefore, it is evident that depression, anxiety, stress, suicidal ideation, post-traumatic stress disorder, major depressive disorder, suicidal tendencies, generalized anxiety disorder and sleep disturbances emerged notably due to natural disasters among people who witnessed natural disasters (Mamun et al., 2021; Siddik et al., 2024; Kabir et al., 2024).

Along with prominent mental health problems, there are some associated risk factors which provokes prominent mental health problems. From the analysis of included studies, it was found that age, gender, living location, living infrastructure, marital status, number of children, educational qualification and gender violence were the major associated risks factors associated with depression, anxiety, stress, GAD, PTSD, suicidal ideation, sleep disturbances following natural disasters (Siddik et al., 2024; Mahmud et al., 2021; Mamun et al., 2019; Hossain et al., 2021a,b; Chandra Das et al., 2022; Moyna et al., 2024; Mostafizur Rahman et al., 2023; Nayna Schwerdtle et al., 2021). For instance, individuals between the age range of 41 and 50 suffered major mental health issues (Chandra Das et al., 2022). PTSD, depression, and psychological distress following flood appear to be prevalent among teenagers (Siddik et al., 2024; Mamun et al., 2019). Women are vulnerable to natural disaster following which 80% girls faced depression due to witnessing natural disasters (Wahid et al., 2023). But, one study found that males reported higher levels of mental health symptoms (Chandra Das et al., 2022). Living disaster prone areas were predicted as significant risk factor of mental health problems. 82.49% of respondents' houses were completely demolished due to erosion who used to live adjacent to embankments (Rahman and Gain, 2020; Mamun et al., 2019). Similarly, marital status especially divorced people, having three or more children showed stress, anxiety, and depression (Hossain et al., 2021b; Kabir et al., 2024). Mamun et al. (2021) found that having less education is associated with developing suicidal behavior. Therefore, based on the present findings, it was précised that those factors enhance the risk of developing prominent mental health problems following natural disasters.

Based on present findings, socio-economic factors including environmental settings, social support, disaster warning system, displacement, food crisis, financial support, income loss, vulnerable livelihoods play key role in developing post-traumatic stress disorder, panic, anxiety, stress, suicidal ideation (Rahman and Gain, 2020; Hossain et al., 2021b; Mamun et al., 2019; Hossain et al., 2021a; Tasdik Hasan et al., 2020; Malak et al., 2020; Mahmud et al., 2021). For example, participants reported higher rates of stress (54.59%), anxiety (95.20%), and depression (63.32%) due to their displacement of living places because of natural disasters (Hossain et al., 2021b), while 40.8% experienced serious shortages of food (Hossain et al., 2021a). Nevertheless, rest of the factors also reported by the rest of the studies significantly. Therefore, it was evident that associated factors assist to increase the risk of developing prominent mental health problems following natural disasters.

Researchers in the US discovered that many Hurricane Katrina survivors suffered from existential anxiety and other mental health issues, including PTSD symptoms and thoughts of suicide. Scott and Weems (2013) used Tillich's (1961) theory of existential anxiety to show that after a tragedy, PTSD symptoms were linked to existential worries about life's emptiness, and that thoughts of suicide were positively correlated with emotions of meaninglessness. In a similar vein, Zhang et al. (2022) found that different socio-demographic characteristics impact the mental health outcomes in crisis situations. They discovered that people with more education showed less emotional reaction to catastrophes than those with less education. Another finding that supports the idea that economic dependence and social connections have a role in mental health outcomes is that middle-aged and older persons who had family members active in agricultural production were more vulnerable to disasters (Eckstein et al., 2021).

In contrast, a study out of Nigeria found that people's intense psychological discomfort after a storm was associated with their feelings of helplessness in the face of their surroundings (Ndanusa and Jonah, 2022). Surprisingly, Rehdanz et al. (2015) found no substantial psychological effects after the Japanese tsunami. Life satisfaction dropped sharply in the impacted regions, but assessments of overall quality of life stayed the same. This finding implies that cultural or contextual factors might impact psychological resilience in the aftermath of a disaster.

From analyzing the included studies, present findings, behavior and health related factors like behavior pattern, physical injury, physical disability during natural disaster significantly related to much greater rates of depression and anxiety (Wahid et al., 2023; Moyna et al., 2024; Mostafizur Rahman et al., 2023; Tasdik Hasan et al., 2020). Having physical injuries in the age range of 18 to 30 years were related to depressive symptoms (Mamun et al., 2019). Similarly, other factors like increased temperature, living together, lower social class are related to depression and anxiety (Mamun et al., 2021; Chandra Das et al., 2022; Wahid et al., 2023).

## 5 Protective mechanisms

It is possible to lessen the impact of natural catastrophes on people's mental health by using resilience factors and protective measures. By bolstering people's and communities' abilities to adapt and recover, protective mechanisms are crucial in preventing negative mental health outcomes. According to research by Norris et al. (2008) and Wind and Komproe (2012), individuals can enhance their psychological well-being by reducing stress and utilizing social support networks, community cohesion, and positive coping mechanisms. Reduced anxiety and despair can be achieved through the power of community connections and the belief in one's own ability to help one another heal (Cutter et al., 2008). Fernando and Hebert (2011) found that those who are financially secure and who hold strong religious or philosophical views are better able to weather difficult times. Interventions to enhance resilience in communities vulnerable to disasters must be based on a thorough understanding of these mechanisms. Such protective mechanisms are listed below:

### 5.1 Social support networks

A well-known protective factor for mental health after disasters is having strong social support. When people have supportive social networks, they are less likely to feel lonely and more likely to bounce back from adversity (Norris et al., 2008). Research has shown that people who are well-connected to their families and communities are less likely to suffer from PTSD and depression in the aftermath of disasters (Bonanno et al., 2007).

### 5.2 Community cohesion and collective efficacy

Collaborative efficacy and community cohesion are especially important in disaster recovery because they promote shared resources and group problem-solving. People feel more empowered and supported when communities come together to address recovery needs, and this builds resilience against mental health issues (Wind and Komproe, 2012). In areas devastated by cyclones, for example, community members' collective efficacy has been linked to a decrease in the prevalence of anxiety and depressed symptoms (Cutter et al., 2008).

### 5.3 Positive coping strategies

In the aftermath of a tragedy, coping mechanisms like problem-solving and emotional control can be quite helpful in maintaining mental health. According to Schnittker (2008), symptoms of anxiety, sadness, and post-traumatic stress disorder (PTSD) are lessened when people employ adaptive coping mechanisms, such as reaching out to social support, being attentive, and keeping hope alive. Individuals' psychological resilience may be enhanced by community-wide instruction and promotion of these tactics, allowing them to better handle the stresses that arise in the aftermath of disasters.

### 5.4 Economic stability and access to resources

To mitigate the psychological effects of catastrophes, it is essential to have access to resources such as food, housing, and financial assistance. Reducing financial instability allows individuals to concentrate on rehabilitation without additional stress, which in turn reduces burden on mental health. Research shows that people with financial security or access to assistance are less likely to suffer from mental health issues compared to those without (Neria et al., 2009).

### 5.5 Cultural and spiritual beliefs

Spiritual beliefs and cultural rituals can offer solace and purpose, particularly in the wake of a tragedy. One way that people deal with and overcome trauma is through cultural narratives, religious practices, and rituals. According to Fernando and Hebert (2011), spiritual beliefs offer a sense of purpose and community belonging, which in turn protects against depression and PTSD.

## 6 Limitations and future research directions

By synthesizing previous studies on mental health problems and their risk factors in the aftermath of natural disasters in Bangladesh, this review fills important knowledge gaps and provides useful insights. A comprehensive review of the state of knowledge in this field can be undertaken primarily to the methodological rigor used to select and analyze papers. It is critical to note, however, the examined study does have some inherent limitations. The principal reasons for our study's limitations are the difficulties in combining different research approaches and situations in the area of mental health following disasters. Our results may not be applicable to a broader population. Although many research used cross-sectional designs, this method severely limits our capacity to make causal conclusions regarding the links between mental health outcomes and risk factors. Moreover, people may be hesitant to disclose mental health problems because of societal constraints or stigma, which means that self-reported assessments could be biased. Another major drawback is that the current literature may not adequately reflect under-represented groups, which could confuse our view of how various demographics deal with mental health difficulties during disasters.

Future studies should aim to fill these gaps and deepen our knowledge of disaster-related mental health issues by concentrating on a few significant areas. Researchers must conduct longitudinal studies in order to track participants' mental health over time and evaluate the changing psychological effects following a disaster. Research on the lived experiences of those impacted must also make use of a variety of methodology, including qualitative techniques. The inclusion of under-represented groups should be a top priority for researchers if they want their results to be generalizable to different settings. Researching the effectiveness of different mental health interventions in disaster-affected populations can also help in creating effective support systems. In the future, researchers can make great strides in the subject and help disaster victims' mental health by following these paths.

## 7 Conclusion

Devastating natural disasters in Bangladesh have a multiplicative effect on the already fragile mental health of the afflicted people, making their conditions even worse. Several important findings about the mental health consequences and related risk factors following such catastrophes have been emphasized in this systematic study. Poor mental health outcomes were consistently predicted by factors such as being female, having a low socioeconomic position, experiencing loss of loved ones, being displaced, and lacking social support. Access to mental health treatments is still restricted, and effective intervention is hindered by stigma and institutional constraints, despite the high burden of mental health concerns post-disaster.

Because of the cumulative effect on already vulnerable communities, natural disasters in Bangladesh have a particularly negative influence on the mental health of the people who experience them. The results of this systematic analysis shed light on the relationship between mental health outcomes and risk variables, which include things like gender, socioeconomic level, displacement, loss of loved ones, and social support. According to these results, which are in line with resilience and existential theories, not only are individual vulnerabilities heightened in the aftermath of a disaster, but community resilience and resource accessibility are also crucial in the healing process. Tragically, stigma, institutional obstacles, and a lack of mental health infrastructure all contribute to a severely underserved population when it comes to mental health treatment. An improved strategy that incorporates trauma-informed treatment and psychological first aid training for healthcare workers and first responders is necessary to address these difficulties within Bangladesh's disaster response systems. Prioritizing community-based psychosocial support programs that stress cultural and contextual relevance might help satisfy the different needs of affected demographics. All members of the community, regardless of cultural background, should be able to participate in these interventions, which should prioritize building stronger communities through increased support networks, more adaptable coping strategies, and stronger community support networks.

Theoretically, this review may benefit from incorporating viewpoints from resilience and social ecology frameworks; specifically, it could investigate the ways in which individual, societal, and environmental elements interact to create mental health outcomes. A more nuanced understanding of mental health resilience and vulnerability could be achieved by more research that investigates opposing ideas, particularly through comparative studies across different areas and disaster kinds. To determine how long mental health symptoms last and what variables lead to recovery, longitudinal studies are mandatory. In conclusion, a thorough, multi-sectoral strategy is necessary to address the mental health issues that arise in the aftermath of natural disasters in Bangladesh. We can help communities recover from disasters and lessen their psychological impact in the long run by including mental health in disaster preparedness and response, building resilience within communities, and increasing access to high-quality care.
