# Supplementary material for: Understanding mental health challenges and associated risk factors of post-natural disasters in Bangladesh: a systematic review
Source: Front Psychol. 2024 Dec 16;15:1466722. doi: 10.3389/fpsyg.2024.1466722 (PMC11682885; doi:10.3389/fpsyg.2024.1466722)
Supplement: Supplementary file 1 [file Data_Sheet_1.PDF]

Supplementary Materials

Supplementary File-01

This table provides a summary of risk levels across studies, which complements the narrative summary by offering specific insight into the risk levels associated with individual studies and domains. This allows readers to understand the strengths and limitations of each study's methodological quality and the overall evidence synthesis.

**Study**	**Selection Bias**	**Performance Bias**	**Detection Bias**	**Reporting Bias**	**Overall Risk Level**
Fatema et al., 2023	Low	Low	Low	Low	Low
Mahmud et al., 2021	Low	Low	Moderate	Moderate	Moderate
Chandra Das et al., 2022	Moderate	High	High	Low	High
Hossain et al., 2021a	Low	Low	Moderate	Low	Moderate
Hossain et al., 2021b	High	Moderate	Moderate	High	High
Mamun et al., 2019	Moderate	Low	Moderate	Moderate	Moderate
Rahman & Gain, 2020	High	High	Moderate	Low	High
Moyna et al., 2024	Moderate	Moderate	High	Moderate	High
Arobi et al., 2020	Low	Moderate	Moderate	Moderate	Moderate
Mostafizur Rahman et al., 2023	High	High	High	Moderate	High
Wahid et al., 2023	Moderate	Low	Low	Moderate	Moderate
Mamun et al., 2021	Low	Low	Moderate	Moderate	Moderate

Study	Selection Bias	Performance Bias	Detection Bias	Reporting Bias	Overall Risk Level
Tasdik Hasan et al., 2020	High	High	High	Moderate	High
Siddik et al., 2024	Low	Moderate	High	High	High
Malak et al., 2020	Moderate	Moderate	High	Moderate	High
Nayna Schwerdtle et al., 2021	Moderate	Low	Low	Moderate	Moderate
Kabir et al., 2024	High	Moderate	Moderate	High	High

Notes

- **Selection Bias**: Risk level is based on randomization and representativeness of the sample.
- **Performance Bias**: Considers blinding and intervention administration across groups.
- **Detection Bias**: Examines consistency and objectivity of outcome assessment.
- **Reporting Bias**: Evaluates completeness and transparency of the data reported.

Supplementary File-02

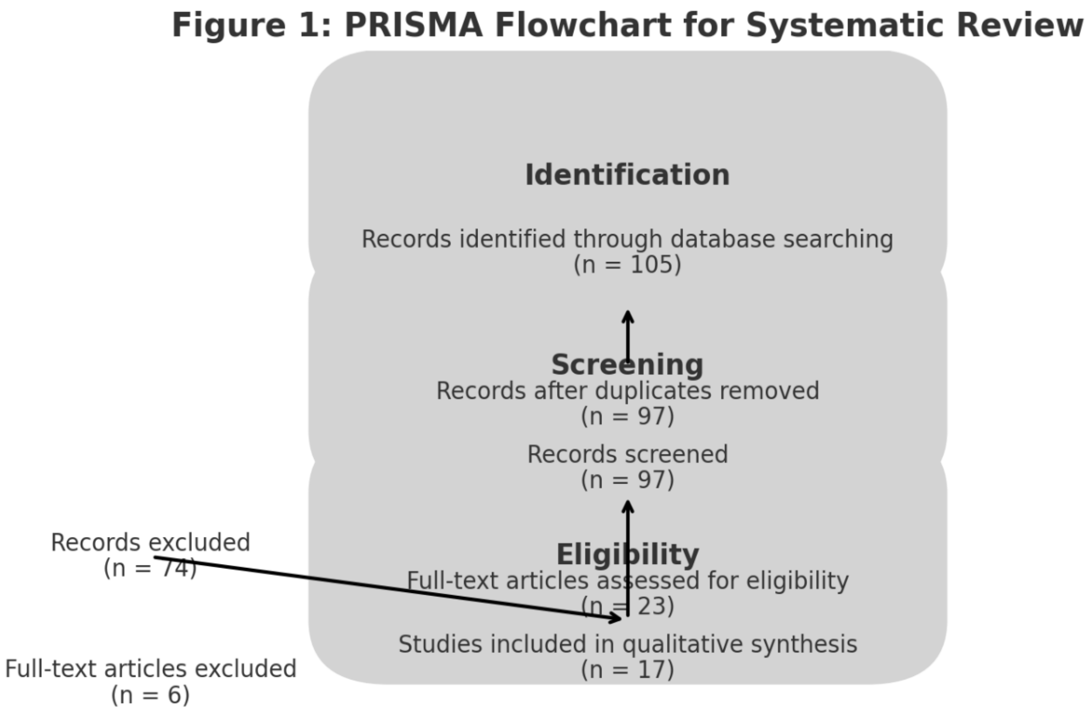

Figure 1: PRISMA Flowchart describing the search strategy and inclusion/exclusion of studies for review (Adapted from Moher *et al.,* 2010).
